# Boost Up Carrier Mobility for Ferroelectric Organic Transistor Memory via Buffering Interfacial Polarization Fluctuation

**DOI:** 10.1038/srep07227

**Published:** 2014-11-27

**Authors:** Huabin Sun, Qijing Wang, Yun Li, Yen-Fu Lin, Yu Wang, Yao Yin, Yong Xu, Chuan Liu, Kazuhito Tsukagoshi, Lijia Pan, Xizhang Wang, Zheng Hu, Yi Shi

**Affiliations:** 1 School of Electronic Science and Engineering, Collaborative Center of Advanced Microstructures, National Laboratory of Solid-State Microstructures, Nanjing University, Nanjing 210093, China; 2 International Centre for Materials Nanoarchitectonics (WPI-MANA), National Institute for Materials Science (NIMS), Tsukuba, Ibaraki 305-0044, Japan; 3 Key Laboratory of Mesoscopic Chemistry of MOE, School of Chemistry and Chemical Engineering, Nanjing University, Nanjing 210093, China

## Abstract

Ferroelectric organic field-effect transistors (Fe-OFETs) have been attractive for a variety of non-volatile memory device applications. One of the critical issues of Fe-OFETs is the improvement of carrier mobility in semiconducting channels. In this article, we propose a novel interfacial buffering method that inserts an ultrathin poly(methyl methacrylate) (PMMA) between ferroelectric polymer and organic semiconductor layers. A high field-effect mobility (*μ*_FET_) up to 4.6 cm^2^ V^−1^ s^−1^ is obtained. Subsequently, the programming process in our Fe-OFETs is mainly dominated by the switching between two ferroelectric polarizations rather than by the mobility-determined charge accumulation at the channel. Thus, the “reading” and “programming” speeds are significantly improved. Investigations show that the polarization fluctuation at semiconductor/insulator interfaces, which affect the charge transport in conducting channels, can be suppressed effectively using our method.

Organic field-effect transistors using poly(vinylidene fluoride-trifluoroethylene) [P(VDF-TrFE)] in its polycrystalline phase as the ferroelectric gate insulator have been the subject of intensive interest for a variety of non-volatile memory device applications^1^,^2^,^3^,^4^,^5^,^6^,^7^,^8^. The improvement in charge carrier mobility of the channel materials is of important issue that determines the development of ferroelectric organic field-effect transistors (Fe-OFETs)^9^,^10^. Organic semiconductors generally exhibit low carrier mobility in such transistor memory devices. Subsequently, the operating speed that regards with the programming and reading processes in a Fe-OFET is bottlenecked by a slow charge accumulation process in the channel, rather than by the rapid molecular reverse between the polarization states of the ferroelectric gate insulator of P(VDF-TrFE)^11^,^12^,^13^. So far, large surface roughness of the polycrystalline P(VDF-TrFE) layer has been considered as the root that significantly affects the charge transport at the semiconductor/insulator interface^14^,^15^,^16^,^17^. Studies have been devoted to the development of methods that reduce the surface roughness of P(VDF-TrFE) and form a smooth semiconductor/insulator interface^18^,^19^. However, the charge carrier mobility is still lower than those acquired using other dielectric layers even with similar surface roughness^20^,^21^. Therefore, it indicates other influences that limit the charge transport behaviour at the semiconductor/ferroelectric interface. Note that a P(VDF-TrFE) film in a polycrystalline phase intrinsically produces a polarization fluctuation at the surface because of the orientation differences of dipole moments among ferroelectric microcrystals^22^. Such a polarization fluctuation can generate a built-in electrical field disturbance at the semiconductor/ferroelectric interface, which affects the charge transport at the channel by inducing additional scattering of charge carriers. Consequently, it is of great interest and importance to suppress the interfacial polarization fluctuation, boosting up the charge carrier mobility at the semiconducting channel, and leading to a high-speed Fe-OFET with the memory device operating processes governed mainly by the switching between polarization states of the ferroelectric insulator.

Herein, we propose a novel interfacial buffering method that deposits an ultrathin polymer of poly(methyl methacrylate) (PMMA) onto the P(VDF-TrFE) layer in bottom-gate Fe-OFETs. Devices with buffered ferroelectric layers exhibit an average and the highest field-effect mobility (*μ*_FET_) of 3.4 and 4.6 cm^2^ V^−1^ s^−1^, respectively. To the best of our knowledge, this is the highest carrier mobility reported so far for Fe-OFETs. Based on the high *μ*_FET_, the programming process in our Fe-OFETs was mainly dominated by the switching between two P(VDF-TrFE) polarization layers rather than by the mobility-determined charge accumulation at the channel. Investigations showed that the ultrathin polymer layer buffered the polarization fluctuation at the semiconductor/insulator interface and enhanced the charge transport at the channel.

## Results

**Figure 1:**
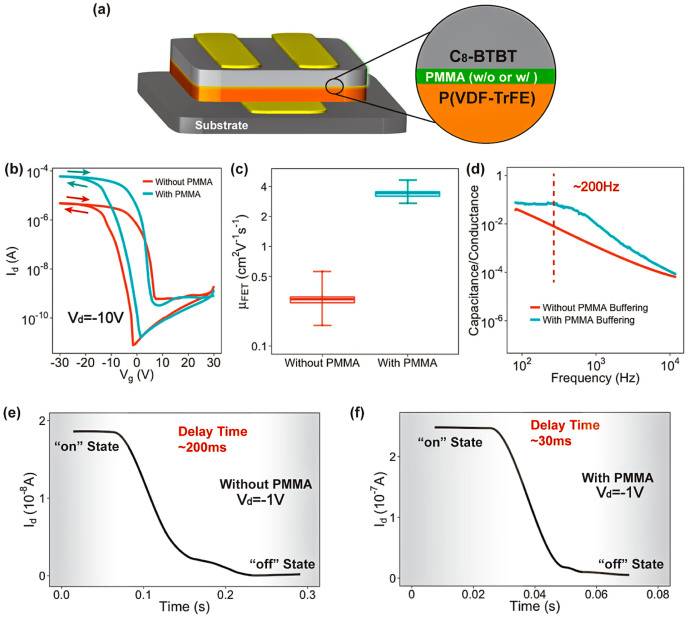
(a) Schematic illustration of a Fe-OFET with a bottom-gate top-contact structure. An ultrathin poly(methyl methacrylate) (PMMA) film acts as a buffering layer between the ferroelectric insulator of poly(vinylidene fluoride-trifluoroethylene) P(VDF-TrFE) and organic semiconductor layers, buffering the polarization fluctuation at the semiconductor/insulator interface. (b) Typical transfer curves of the devices without (red line) and with (blue line) PMMA buffering layer. (c) Distributions of the field-effect mobility (*μ*_FET_) of devices without and with PMMA buffering. (d) Dependence of capacitance divided by the channel conductance on the gate voltage frequency. (e) and (f) present the pulse responses of the Fe-OFETs without and with PMMA buffering, respectively.

We initially fabricated Fe-OFET devices without the buffering method for the P(VDF-TrFE) layers. Dioctylbenzothienobenzothiophene (C_8_-BTBT), a p-type small-molecule semiconductor, was thermally evaporated to form a semiconductor channel in a bottom-gate top-contact architecture (Fig. 1a). The red line in Fig. 1b represents a transfer curve of the device with *μ*_FET_ of 0.55 cm^2^ V^−1^ s^−1^. And an average *μ*_FET_ calculated from seven devices is 0.32 cm^2^ V^−1^ s^−1^ (Fig. 1c). This low *μ*_FET_ leads to a relatively slow charge accumulation in response to the gate voltage^13^,^23^. We performed a frequency response to represent the “reading” process (Fig. 1d). The capacitance of gate insulator is divided by the channel conductance, in order to avoid the influence of gate leakage current^21^. The device without PMMA buffering shows a slow “reading” speed beyond measurement limits (frequency <20 Hz, “reading” time > 50 ms). Furthermore, the pulse response of the Fe-OFET, which represents the “programming” process, presents a large delay time value from high- to low-conductance states of more than 200 ms (Figs. 1e and S1). In comparison, devices using PMMA buffering layers exhibit significant performance enhancement. The blue line in Fig. 1b represents a transfer curve of the device. It exhibits the highest *μ*_FET_ of 4.6 cm^2^ V^−1^ s^−1^, which is the highest carrier mobility reported so far for Fe-OFETs (Table S1). And the transfer curves with a low gate voltage range (−2 V to 2 V) after writing and erasing indicate a build-in electric field after writing and erasing in the devices (Fig. S2). The average *μ*_FET_ value of the seven devices is 3.4 cm^2^ V^−1^ s^−1^, which is one magnitude order larger than that of devices without the buffering process (Fig. 1c). Besides, a slight change in the memory window from 12 V to 10 V was observed after buffering (Figs. 1b and S3). Furthermore, the enhanced carrier mobility in the semiconducting channel further improved operation speed, which is an important parameter that evaluates the Fe-OFET performance memories. Fig. 1d shows that the curve of frequency response starts to decrease from 200 Hz, indicating a reading time of <5 ms. Moreover, as shown in Fig. 1f, the abrupt switching of the drain current reveals a quick pulse response to the gate voltage pulse, indicating a short delay time in the drain current of ~30 ms. This delay time is close to the switching time between two polarizations of P(VDF-TrFE) under the same bias^24^. Therefore, the ferroelectric insulator mainly controls the programming speed of buffered Fe-OFETs. Besides, devices using PMMA buffering also yield an improved retention capability (Fig. S4). Since the electrical characteristics were measured under ambient air conditions, the obtained retention performance also indicates a good stability of our Fe-OFETs. Furthermore, a higher *μ*_FET_ of 7.2 cm^2^ V^−1^ s^−1^ was obtained from devices with thicker PMMA layers (Fig. S5), while the memory effect is substantially weakened because of the strong screening effect of the PMMA at the insulator surface^25^.

## Discussion

**Figure 2:**
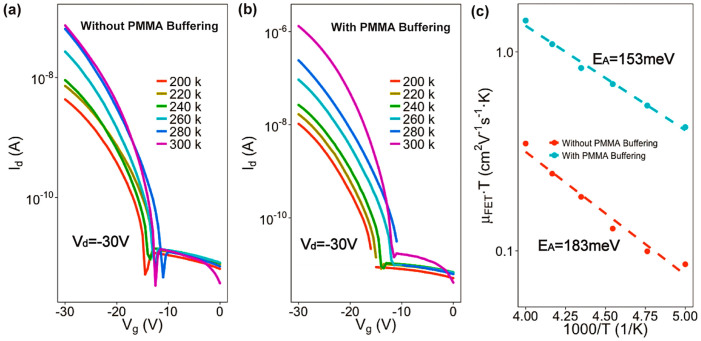
Transfer curves of typical devices without (a) and with (b) PMMA buffering under different temperatures (*T*). (c) shows that *μ*_FET_ calculated from the transfer curves in (a) and (b) both can be well fitted to a straight lines in the plot of *μ*_FET_**·***T* versus 1/*T*. And activation energies of 183 meV and 153 meV for the devices without and with PMMA buffering are calculated, respectively.

The measurement of transfer curves under a range of temperatures was further studied to determine the intrinsic effect of our buffering method. In order to circumvent influence of the memory effect, we deposited P(VDF-TrFE) onto the surface of a 100 nm thick SiO_2_ (Fig. S6). Fig. 2a and 2b show that the current flowing from source to drain electrodes in devices with and without buffering layers both decreases when the temperature is lowered. Fig. 2c shows that the extracted activation energies of the devices with and without PMMA buffering are 183 meV and 153 meV, respectively. These results indicate that our buffering method can lead to a smoother charge transport at the semiconductor/insulator interface simply by depositing a PMMA layer on the P(VDF-TrFE) surface^26^,^27^. The polymeric dielectric of PMMA, which has strong polarity in nature, can interacts with organic semiconductors at interfaces. It forms interfacial polarons that lead to high activation energy and inferior charge transport at conducting channel^22^,^27^,^28^,^29^. However, our buffering method exhibited the opposite behaviour. Covering the P(VDF-TrFE) surface with PMMA resulted in reduced activation energy.

**Figure 3:**
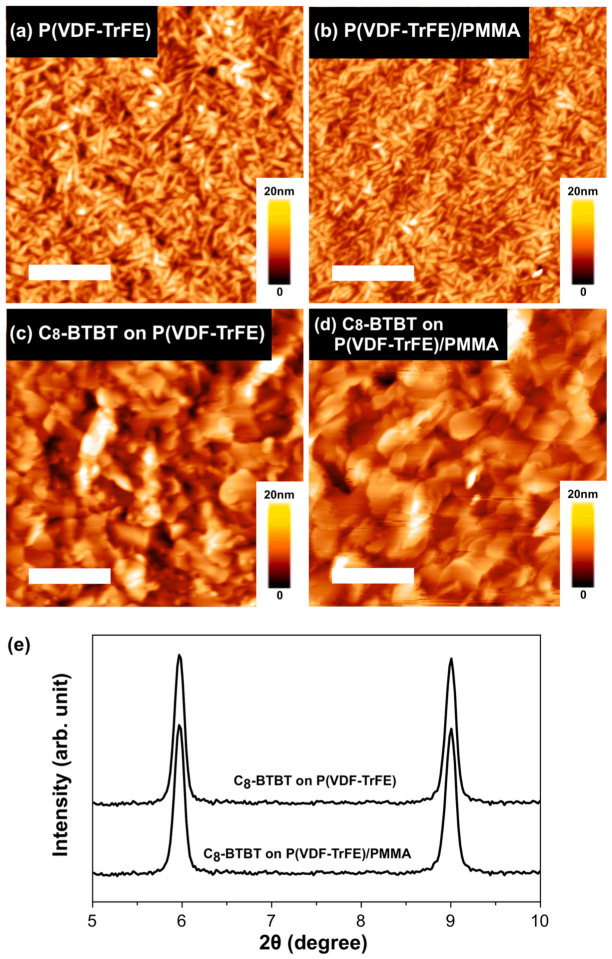
Atomic force microscopy (AFM) images of the surfaces of (a) P(VDF-TrFE), (b) P(VDF-TrFE)/PMMA, (c) C_8_-BTBT film on P(VDF-TrFE), and (d) C_8_-BTBT film on P(VDF-TrFE)/PMMA. The scale bars in a) to d) are for 500 nm in length. (e) X-ray diffraction (XRD) signals of C_8_-BTBT films on P(VDF-TrFE) and P(VDF-TrFE)/PMMA.

Reduction in the activation energy normally results from improved crystalline property of organic semiconductors and optimized interface properties. We thermally evaporated the organic semiconductor onto the P(VDF-TrFE) layers with and without buffering to preclude the influence of crystalline properties of the semiconductor layer. Firstly, surfaces of P(VDF-TrFE) layers without and with PMMA buffering were examined using atomic force microscopy (AFM). As shown in Fig. 3a and 3b, both exhibit as polycrystalline films with similar needle-like domains and roughness. Furthermore, Fig. 3c and 3d show the topographic AFM images of the C_8_-BTBT layers on different gate insulators. Both C_8_-BTBT layers exhibited nearly identical roughness and domain size. Additionally, the x-ray diffraction (XRD) results show nearly identical peak positions and full widths at half maximum for C_8_-BTBT films on P(VDF-TrFE) layers without and with buffering (Fig. 3e). It confirms the similarity of the crystalline properties, implying that the molecular packing and domain sizes in both films are almost the same.

**Figure 4:**
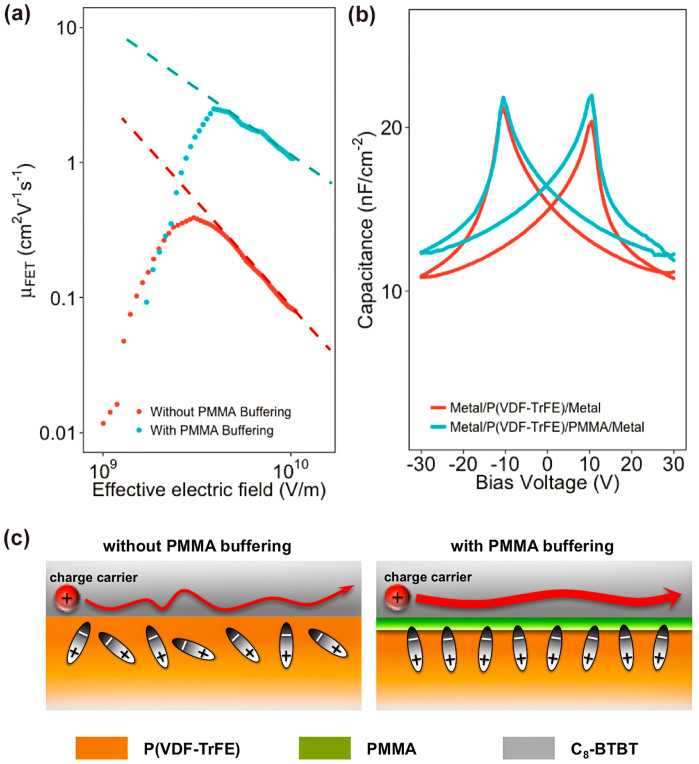
(a) Dependence of *μ*_FET_ for device without (red circles) and with (blue circles) on the electric field that effectively controls the charge carriers in channel. (b) Capacitance versus bias voltage results for the metal-insulator-metal samples with different insulator layers of P(VDF-TrFE) (red line) and P(VDF-TrFE)/PMMA (blue line). (c) (left) Illustrative representation of the polarization fluctuation at the semiconductor/insulator interface, affecting the charge carrier transport. (right) Such a polarization fluctuation can be well supressed by depositing an ultrathin PMMA film between the ferroelectric and semiconductor layers, and the charge carrier transport is enhanced.

Consequently, the significant improvement in Fe-OFETs performance by using PMMA buffering has negligible correlation with the crystalline property of organic semiconductor. Also, the AFM results demonstrate that the P(VDF-TrFE) layers without and with buffering both exhibit similar polycrystalline morphologies (Fig. 3a and 3b). And surface roughness has a negligible difference: the RMS roughness of bare P(VDF-TrFE) is 2.46 nm, whereas that of the buffered one is 2.36 nm. However, different slopes of fitting lines are observed in the plot that presents the dependence between μ_FET_ of our Fe-OFETs and the effective electric field (*E*_eff_) controlling the charge carriers at channel (Fig. 4a). Such dependence indicates a charge transport dominated by surface scattering^30^,^31^, 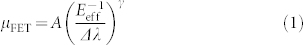
where *A* is a constant, *Δ* is the surface roughness, *λ* is the correlation length, and *γ* is related to the interface properties (excluding interface roughness). The slope of the fitting line represents *γ*. A polycrystalline P(VDF-TrFE) film can intrinsically produce a polarization fluctuation at surface, subsequently building an electrical field disturbance at the semiconductor/ferroelectric interface. Thus, the decrease in the slope as observed from the Fe-OFET with buffering indicates that the coverage of an ultrathin PMMA layer optimizes the semiconductor/insulator interface by suppressing the polarization fluctuation, resulting in a smooth charge transport at the conducting channel. Furthermore, the values of *γ* were extracted from the relationship between *μ*_FET_ and *E*_eff_. We observed a linear relationship between ln(*μ*_FET_) versus *γ* under the same *E*_eff_ (Fig. S7). Note that *γ* represents the interface properties, and the change in *γ* is due to the PMMA buffering at the semiconductor/ferroelectric interface. Therefore, the linear relationship between ln(*μ*_FET_) versus *γ* confirms that the significant enhancement in *μ*_FET_ mainly results from the PMMA buffering of the interfacial polarization fluctuation. Besides, since the activation energy associates with the hopping transport of charge carriers in organic semiconductors, the reduced activation energy in the Fe-OFETs with PMMA buffering is also attributed to the suppression of the polarization fluctuation at the semiconductor/ferroelectric interface.

Capacitance versus voltage measurements were performed on parallel-plate capacitors with metal–insulator–metal (MIM) structures to further understand the PMMA influence. As shown in Fig. 4b, MIM samples using P(VDF-TrFE) without and with PMMA both exhibit hysteresis with a butterfly shape. It stems from the irreversible ferroelectric polarization, that is, dipole rotation in the presence of a bias voltage. A higher capacitance is obtained in the sample with a PMMA layer than the one without. Note that polycrystalline P(VDF-TrFE) films naturally have a polarization fluctuation at surface. Higher capacitance in the sample with PMMA-buffered P(VDF-TrFE) indicates a suppression of such a polarization fluctuation by weakening orientation differences of dipole moments among ferroelectric microcrystals. In the case of Fe-OFETs using PMMA, it produces a uniform electrical field at the semiconductor/insulator interface with buffered polarization fluctuation, which is favorable for a smooth charge transport at the conducting channel (Fig. 4c). Also, a control sample using SiO_2_/PMMA as the gate insulator exhibited a *μ*_FET_ of 3.5 cm^2^ V^−1^ s^−1^, which is in the same range of charge mobility as obtained in the Fe-OFETs with PMMA buffering (Fig. S8). And it confirms that our buffering method can efficiently screen the influence of the polarization fluctuation on the charge transport at the PMMA surface. Besides, further increase in PMMA thickness leads to a reduced capacitance value in the MIM sample, where the thick PMMA acts mainly as a series capacitor (Fig. S9). Moreover, this suppression of polarization fluctuation through our buffering method can be devoted to the interfacial interactions between the carbonyl groups of PMMA and the CH_2_ groups of P(VDF-TrFE), which allows well-organized P(VDF-TrFE) molecules at the interface^32^. It thus results in an ordered build-in ferroelectric filed near the interface. Such a study is underway in our laboratory.

In conclusion, we demonstrated an effective buffering method using an ultrathin PMMA layer on ferroelectric dielectric of P(VDF-TrFE) for high-performance Fe-OFETs. A high mobility of up to 4.6 cm^2^ V^−1^ s^−1^ was obtained. Based on the high *μ*_FET_, the programming process in our Fe-OFETs was mainly dominated by switching between two P(VDF-TrFE) polarizations. This enhanced performance was attributed to suppression of the polarization fluctuation at the semiconductor/insulator interface. This study represents a major step in Fe-OFET development. It also reveals that the polarization fluctuation at semiconductor/insulator interfaces, which affect the charge transport in conducting channels, can be buffered effectively using our method.

## Methods

### Device fabrication

For Fe-OFET fabrication, highly doped n-type (100) silicon wafers with 50 nm SiO_2_ layers were first cleaned in an ultrasonic bath in a succession of acetone and 2-propanol for 10 min each. Bottom gate electrodes were formed through thermal evaporation of Ti (3 nm) and Au (20 nm) at a deposition speed of ~0.1 Å/s. P(VDF-TrFE) (Solvay, Inc.) was dissolved in 2-butanone with 3 wt.% concentration. The P(VDF-TrFE) solution was spin-coated onto the Si/SiO_2_ substrate, which was then soft-baked at 90°C for 30 min. The substrate was exposed to an annealing process in a vacuum chamber at 120°C for 2 h. For the buffering process, PMMA (Sigma-Aldrich MW = 996 k) was dissolved in anisole (Sigma-Aldrich) with a concentration of 0.1 wt.%. The solution was spin-coated onto the P(VDF-TrFE) film, followed by soft backing at 90°C for 10 min. C_8_-BTBT was thermally evaporated at a speed of ~0.1 Å/s to obtain 30 nm-thick semiconducting films. Finally, MoO_3_ (3 nm) and Au (35 nm) were successively evaporated under the same conditions to form the source and drain electrodes. And the channel width and length were 1000 μm and 200 μm, respectively.

### Film characterizations

The AFM measurements were performed through AFM using an SPA-400 scanning probe microscope controlled by SPI (4000 probe station, Seiko Instruments, Inc.). X-ray diffraction results were collected using D/MAX-2000/PC (Rigaku Corporation, CuKa).

### Device characterizations

Electrical performance was characterized under ambient conditions using an Agilent 4156C semiconductor parameter analyzer. And device characteristics under a range of temperatures were measured in a vacuum chamber (<3 × 10^−4^ Pa). The Agilent 4980a LCR parameter analyzer was applied in the C–V measurements. The cooling process was set to as low as 0.2 K/min during the temperature-dependent process.

## Author Contributions

H.B.S., Y.L., Y.S. and K.T. conceived and designed the research. H.B.S., Y.L. and Q.J.W. conducted the experiments. All authors participated in the analyses, but Y.F.L., Y.X. and C.L. conducted most of the device performance analyses, and Y.W., Y.Y. and L.J.P. conducted most of the AFM analyses. X.Z.W. and Z.H. contributed to the discussion on the operating mechanism of Fe-OFET memory. H.B.S. and Y.L. wrote the manuscript and prepared the figures. All authors reviewed the manuscript.

## Supplementary Material

Supplementary Information supplementary information
