# Supplementary material for: Boost Up Carrier Mobility for Ferroelectric Organic Transistor Memory via Buffering Interfacial Polarization Fluctuation
Source: Sci Rep. 2014 Nov 27;4:7227. doi: 10.1038/srep07227 (PMC4245676; doi:10.1038/srep07227)
Supplement: Supplementary Information [file srep07227-s1.pdf]

Supplementary Information

Boost Up Carrier Mobility for High-Speed Ferroelectric Organic Field-Effect Transistor Memory via Buffering Interfacial Polarization Fluctuation

Huabin Sun, Qijing Wang, Yun Li*, Yen-Fu Lin, Yu Wang, Yao Yin, Yong Xu, Chuan Liu, Kazuhito Tsukagoshi*, Lijia Pan, Xizhang Wang, Zheng Hu, and Yi Shi*

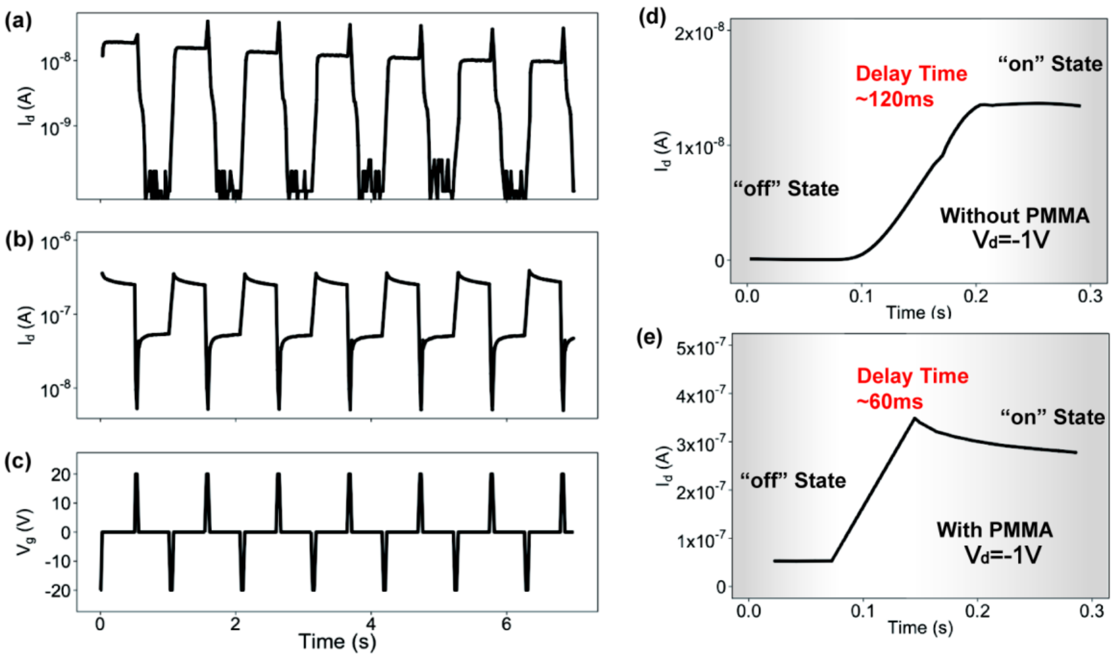

Figure S1. Programming cycles of Fe-OFETs a) without and b) with PMMA buffering layer. c) Gate voltage pulses used to perform the programming processing. (d) and (e) present the pulse responses of the Fe-OFETs from "off" to "on" without and with PMMA buffering, respectively.

Table S1. Performances of typical Fe-OFETs reported in literature.

Ref.	Channel Materials	Ferroelectric	Device Structure	μ_{FET} ($cm^2\ V^{-1}\ s^{-1}$)	On/Off Ratio (V_g = 0 V)	Retention (s)
1	pentacene	P(VDF-TrFE)	BGTC	0.12	10^2	10^5
2	pentacene	P(VDF-TrFE)	BGTC	0.18	20	10^5
3	TIPS-PEN (single crystals)	P(VDF-TrFE)	BGTC	0.29	5×10^2	5×10^5
4	TIPS-PEN (single crystals)	PVDF/PMMA (blend solution)	BGTC	0.65	5×10^2	5×10^5
5	MEH-PPV	P(VDF-TrFE)	BGBC	1.3×10^{-3}		
6	rubrene (single crystals)	P(VDF-TrFE)	BGTC	0.71	2.4	
7	QQT(CN)4	P(VDF-TrFE)	BGTC	0.1	5×10^3 (V_g = −10 V)	6×10^3
8	PC12TV12T	P(VDF-TrFE)	TGBC	0.1	10	10^3
9	P3HT (nanowires)	P(VDF-TrFE)	BCTG	0.01	10 (V_g = −10 V)	10^5
10	P3HT	P(VDF-TrFE)	BCTG	0.12		
this work	C_8-BTBT	P(VDF-TrFE) (with PMMA buffering)	BGTC	4.6	10^4	4×10^3

*TIP-PEN: 6,13-bis(triisopropylsilylethynyl)pentacene. MEH-PPV: poly(2-methoxy-5-(2'-ethylhexoxy)-1,4-phenylene vinylene). QQT(CN)4: quinoidal oligothiophene derivative. PC12TV12T: dodecyl-substituted thienylenevinylene-thiophene copolymer. P3HT: poly(3-hexylthiophene). BGTC: bottom-gate top-contact. BGBC: bottom-gate bottom-contact. TGBC: top-gate bottom-contact.

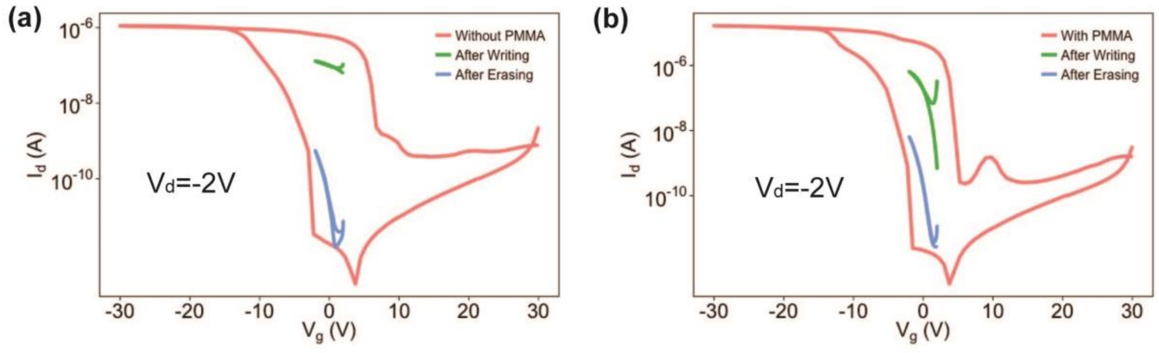

Figure S2. The transfer curves at a low gate voltage range (−2 V to 2 V) after writing and erasing.

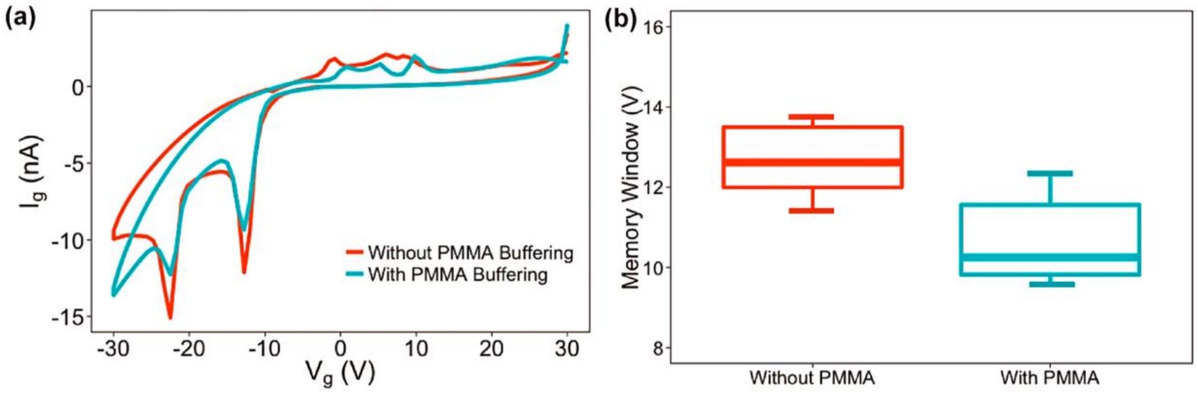

Figure S3. (a) Gate leakage current of Fe-OFETs without (red line) and with (blue line) poly(methyl methacrylate) (PMMA) buffering layers. (b) Distributions of memory windows of Fe-OFETs without and with PMMA buffering layers.

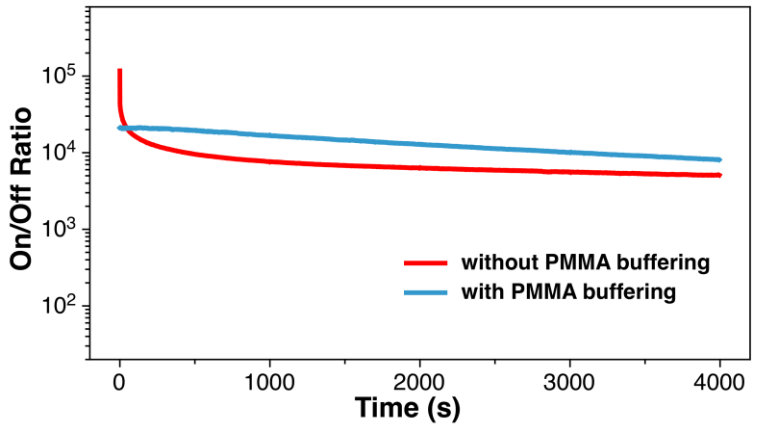

Figure S4. Retention measurements of the Fe-OFETs without (red line) and with (blue line) PMMA buffering layer. Although the device without PMMA initially yields a high on/off ratio of ~10^5, the on/off ratio decreases to 10^4 during a short time of <500 s. And the Fe-OFET with PMMA buffering shows an improved retention capability, exhibiting a much longer time (>3000 s) during which the on/off ratio keeps a value of >10^4.

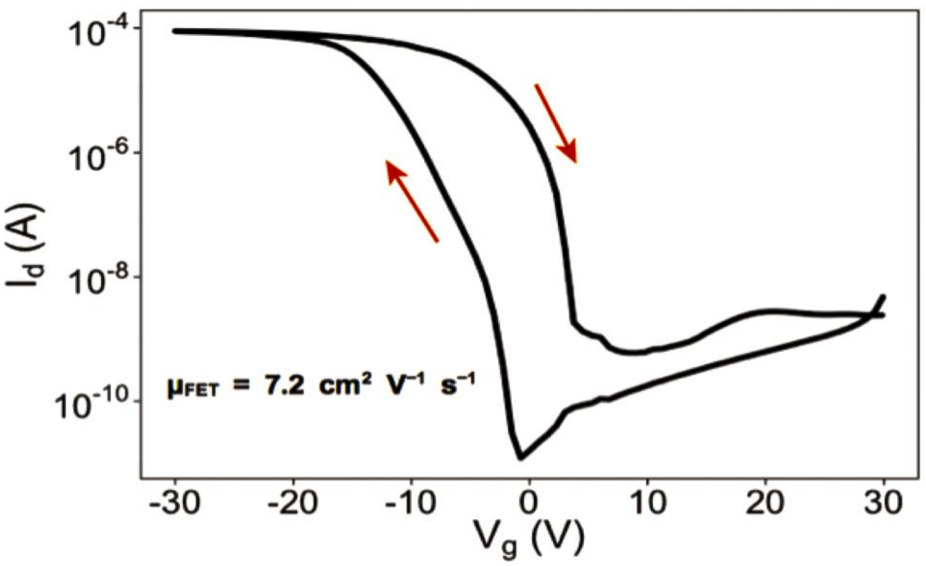

Figure S5. Transfer curve of a Fe-OFET using thicker PMMA buffering layer, exhibiting a high field-effect mobility (μ_{FET}) of 7.2 cm^2 V^{-1} s^{-1} (Vd = −10V).

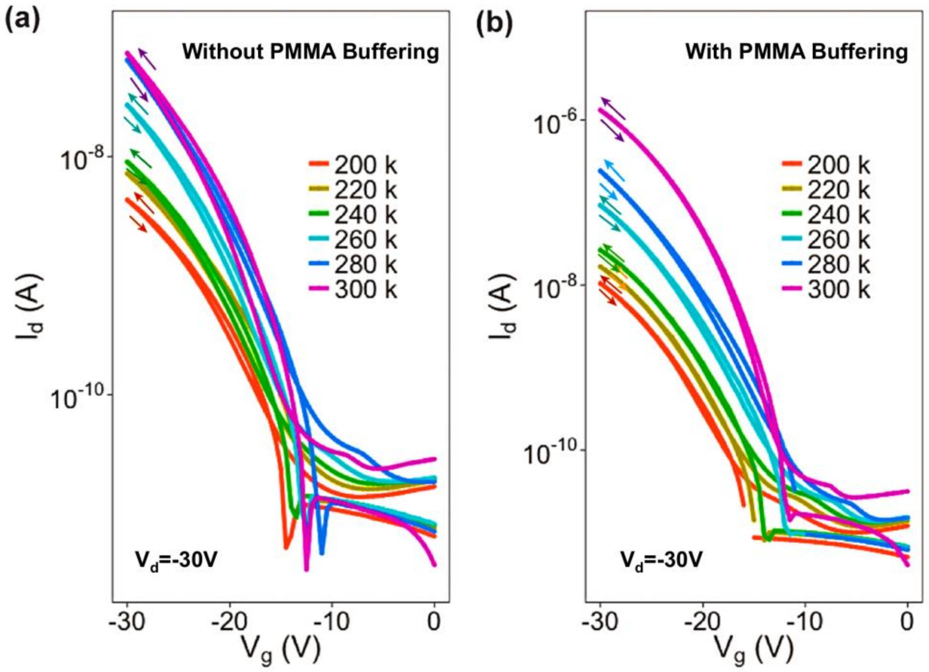

Figure S6. Transfer curves of typical devices without (a) and with (b) PMMA buffering under different temperatures (T). The arrows show the sweep directions, exhibiting anti-clockwise hysteresis.

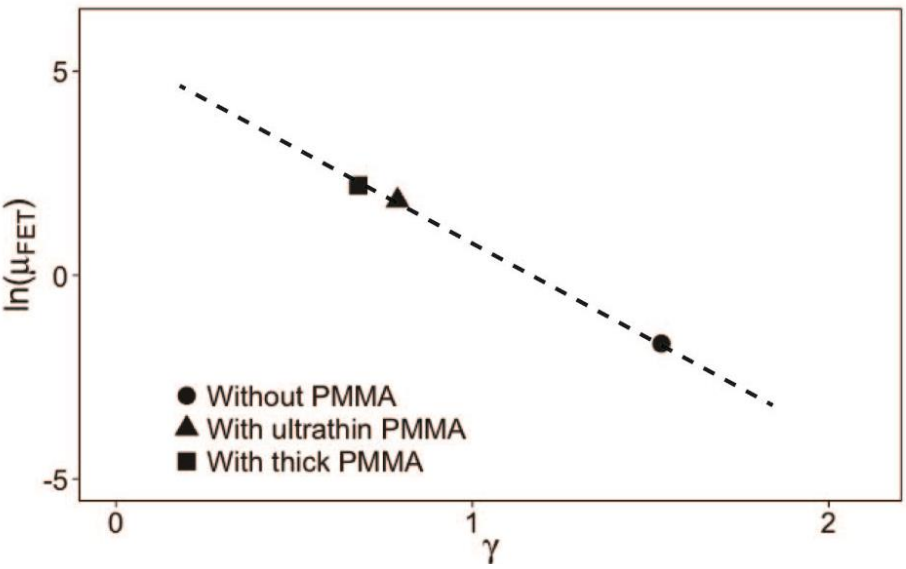

Figure S7. The relationship between μ_{FET} and γ under the same effective electric field. The dash line in the figure is guided by eye.

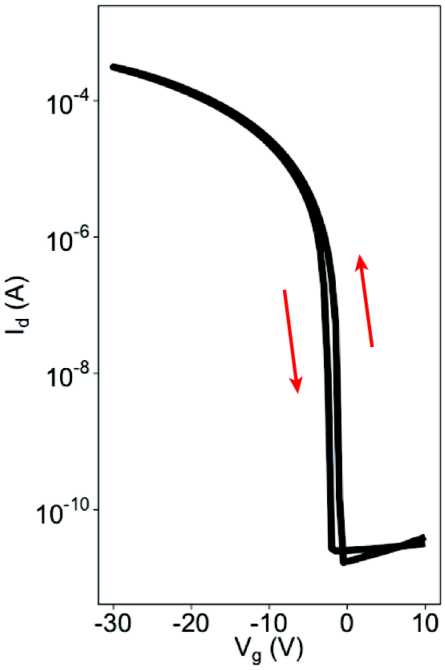

Figure S8. Transfer curve of the control sample using SiO_2 (100 nm)/PMMA (~10 nm) as the gate insulator. The value of μ_{FET} is 3.5 cm^2 V^{-1} s^{-1} (V_d = −30 V), which is in the same range of that obtained from the Fe-OFETs with PMMA buffering.

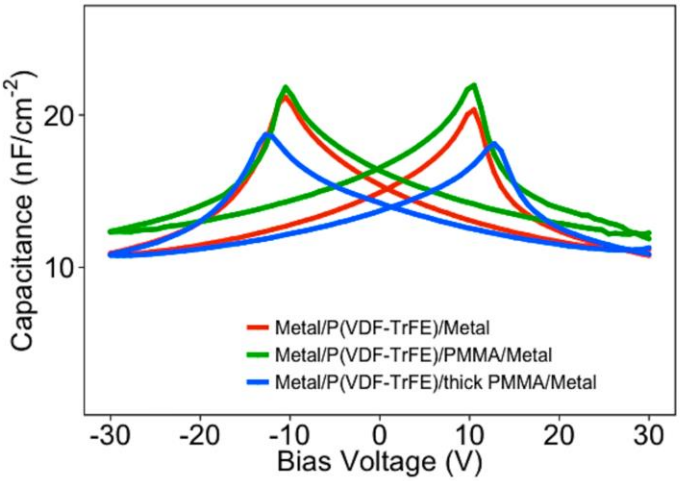

Figure S9. Capacitance (C) versus bias voltage (V) measurements of the metal-insulator-metal samples with different insulator layers of P(VDF-TrFE) (red line), P(VDF-TrFE)/PMMA (thin) (green line), and P(VDF-TrFE)/PMMA (thick) (blue line).

References

1. Khan, M. A., Bhansali, U. S. & Alshareef, H. N. High-Performance Non-Volatile Organic Ferroelectric Memory on Banknotes. *Adv. Mater.* **24,** 2165–2170 (2012).

2. Lee, K. H., Lee, G., Lee, K., Oh, M. S., & Im, S. Flexible low voltage nonvolatile memory transistors with pentacene channel and ferroelectric polymer. *Applied Physics Letters* **94**, 093304 (2009).

3. Kang, S. J., Bae, I., Park, Y. J., Park, T. H., Sung, J., Yoon, S. C. & Park, C. Non‐volatile Ferroelectric Poly (vinylidene fluoride‐co‐trifluoroethylene) Memory Based on a Single‐Crystalline Tri‐isopropylsilylethynyl Pentacene Field‐Effect Transistor. *Advanced Functional Materials* **19**, 1609-1616 (2009).

4. Kang, S. J., Park, Y. J., Bae, I., Kim, K. J., Kim, H. C., Bauer, S. & Park, C. Printable Ferroelectric PVDF/PMMA Blend Films with Ultralow Roughness for Low Voltage Non‐Volatile Polymer Memory. *Advanced Functional Materials*, **19**, 2812-2818 (2009).

5. Naber, R. C. G. *et al.* High-performance solution-processed polymer ferroelectric field-effect transistors. *Nature Materials* **4,** 243–248 (2005).

6. Kanashima, T., Yabe, K. & Okuyama, M. Organic Ferroelectric Field-Effect Transistor Memory Using Flat Poly(vinylidene fluoride–tetrafluoroethylene) and Pentacene Thin Films. *Jpn. J. Appl. Phys.* **51,** 02BK06 (2012).

7. Kim, R. H. *et al.* Non-volatile organic memory with sub-millimetre bending radius. *Nat Comms* **5,** 1–12 (2014).

8. Jung, S. W., Na, B. S., Baeg, K. J., Kim, M., Yoon, S. M., Kim, J. & You, I. K. Nonvolatile Ferroelectric P (VDF-TrFE) Memory Transistors Based on Inkjet-Printed Organic Semiconductor. *ETRI Journal* **35**, 734-737 (2013).

9. Hwang, S. K., Min, S. Y., Bae, I., Cho, S. M., Kim, K. L., Lee, T. W. & Park, C.. Non‐Volatile Ferroelectric Memory with Position‐Addressable Polymer Semiconducting Nanowire. *Small* **10**, 1976-1984 (2014).

10. Naber, R. C. G., Mulder, M., De Boer, B., Blom, P. W. M. & de Leeuw, D. M. High charge density and mobility in poly(3-hexylthiophene) using a polarizable gate dielectric. *Organic Electronics* **7,** 132–136 (2006).
